# Automated Detection of Healthcare Associated Infections: External Validation and Updating of a Model for Surveillance of Drain-Related Meningitis

**DOI:** 10.1371/journal.pone.0051509

**Published:** 2012-12-07

**Authors:** Maaike S. M. van Mourik, Karel G. M. Moons, Wouter W. van Solinge, Jan-Willem Berkelbach-van der Sprenkel, Luca Regli, Annet Troelstra, Marc J. M. Bonten

**Affiliations:** 1 Department of Medical Microbiology, University Medical Utrecht, Utrecht, The Netherlands; 2 Julius Center for Health Sciences and Primary Care, University Medical Utrecht, Utrecht, The Netherlands; 3 Department of Clinical Chemistry and Hematology, University Medical Utrecht, Utrecht, The Netherlands; 4 Department of Neurosurgery, Rudolf Magnus Institute of Neuroscience, University Medical Utrecht, Utrecht, The Netherlands; New York State Health Department and University at Albany, United States of America

## Abstract

**Objective:**

Automated surveillance of healthcare-associated infections can improve efficiency and reliability of surveillance. The aim was to validate and update a previously developed multivariable prediction model for the detection of drain-related meningitis (DRM).

**Design:**

Retrospective cohort study using traditional surveillance by infection control professionals as reference standard.

**Patients:**

Patients receiving an external cerebrospinal fluid drain, either ventricular (EVD) or lumbar (ELD) in a tertiary medical care center. Children, patients with simultaneous drains, <1 day of follow-up or pre-existing meningitis were excluded leaving 105 patients in validation set (2010–2011) and 653 in updating set (2004–2011).

**Methods:**

For validation, the original model was applied. Discrimination, classification and calibration were assessed. For updating, data from all available years was used to optimally re-estimate coefficients and determine whether extension with new predictors is necessary. The updated model was validated and adjusted for optimism (overfitting) using bootstrapping techniques.

**Results:**

In model validation, the rate of DRM was 17.4/1000 days at risk. All cases were detected by the model. The area under the ROC curve was 0.951. The positive predictive value was 58.8% (95% CI 40.7–75.4) and calibration was good. The revised model also includes Gram stain results. Area under the ROC curve after correction for optimism was 0.963 (95% CI 0.953– 0.974). Group-level prediction was adequate.

**Conclusions:**

The previously developed multivariable prediction model maintains discriminatory power and calibration in an independent patient population. The updated model incorporates all available data and performs well, also after elaborate adjustment for optimism.

## Introduction

Surveillance and feedback of healthcare-associated infection (HAI) rates to healthcare workers is considered a cornerstone of infection prevention programs [1], [2]. Policy makers and the public increasingly demand transparent reporting of infection rates to quantify quality of healthcare, for example through surveillance networks such as the National Healthcare Safety Network (NHSN) in the United States or the PREZIES network in the Netherlands [3]–[6]. Because of the potential impact of HAI rates on healthcare utilization and reimbursement, the development of efficient and reliable surveillance methods is of increasing importance. In many circumstances, manual chart review of all patients is still the only available method for surveillance, although it is prone to error due to effort dependent case-finding and the possibility of inconsistent interpretation of case definitions [7], [8]. Possibilities for automated surveillance of HAI using a variety of data sources have been investigated over the past two decades with varying success [9].

A HAI for which routine surveillance is implemented in our institution is drain-related meningitis (DRM), a relatively frequent complication of the use of external ventricular (EVD) and lumbar (ELD) cerebrospinal fluid drains in neurosurgical patients. DRM rates range from 2 up to 25% per drain placed [10]–[12] or 7.5 to 32 infections per 1000 days at risk (DAR) [13]–[15]. Causative micro-organisms are often skin flora, such as coagulase-negative staphylococci and *Staphylococcus aureus*, although in some settings Gram-negative micro-organisms (eg enterobacteriaceae) play an important role [11], [13]. Infection rates also depend on the definition applied. Since surveillance aims to generate insight into rates and characteristics of DRM, definitions are not necessarily identical to a clinical diagnosis entailing treatment consequences. Importantly, some case-definitions, including the CDC-definition for healthcare-associated meningitis, allow for diagnosis of an infection without the presence of bacterial growth from clinical cultures [16], [17].

**Figure 1:**
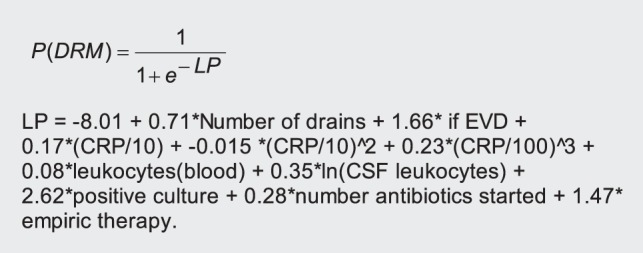
Previously derived prediction rule for drain-related meningitis. For each individual patient, the model returns a predicted probability of DRM which can be used to classify patients. Abbreviations: P(DRM) – probability of drain-related meningitis, LP – linear predictor, EVD – external ventricular drain, CRP – C-reactive protein, CSF – cerebrospinal fluid.

Recently, an accurate prediction model for the automated surveillance of DRM has been proposed which combines predictors from multiple sources to identify those patients which have a high probability of having developed DRM during their admission, both cases of DRM with and without documented pathogens in microbiological cultures (**Figure** **1**) [14]. Such a model can provide more timely and reliable rates of DRM and manual chart review can then be limited to high-risk patients (with a high predicted probability of DRM) while maintaining sensitivity of detection. Importantly, the predictors are all collected during routine clinical care which facilitates applicability of the model in practice [18].

Prediction models require validation in independent patient populations to assess their validity and performance in future use [19], [20]. This research presents the temporal validation of the DRM prediction model. Besides validation, optimal model performance in future patients can be achieved by updating the model using both derivation and validation data [21], [22]. Several newly available predictors were also considered in model updating.

## Methods

### Ethics statement

As described previously, use of anonymized data from the clinical data warehouse has been exempted from review by the institutional review board of our institution [23].

### Development study details

For details on model development, please refer to [14]. In brief, logistic regression was used to develop a prediction model aimed at identifying patients that developed DRM after placement of an EVD or ELD. The study was conducted at University Medical Center Utrecht, a 1042-bed tertiary medical center. Patients who entered the routine surveillance performed by the department of hospital hygiene and infection control between January 1^st^ 2004 and December 31^st^ 2009 were included, with the exception of children, patients with less than one day of follow-up, patients with known meningitis at the time of placement of the first drain, patients admitted with a drain *in situ* or multiple simultaneous drains, military personnel and multiple (independent) admissions within the study period (n = 537 in analysis). All EVDs were placed in operating theatres and are tunneled five centimeters under the skin. Drains were not coated with antibiotics and all patients received perioperative antibiotic prophylaxis. ELDs were either inserted in the operating theatres or in sterile conditions on the neurology ward. Drains were not exchanged on a prophylactic basis and CSF samples were collected for culture and biochemical analysis only when infection was clinically suspected. Clinical care data were obtained from the Utrecht Patient Oriented Database (UPOD), a clinical data warehouse developed for research purposes which links patient characteristics to results from clinical chemistry and medical microbiology laboratories and pharmacy records [23]. Missing data were imputed using multiple imputation, and internal validation was performed [24], [25].

### Outcome

**Figure 2:**
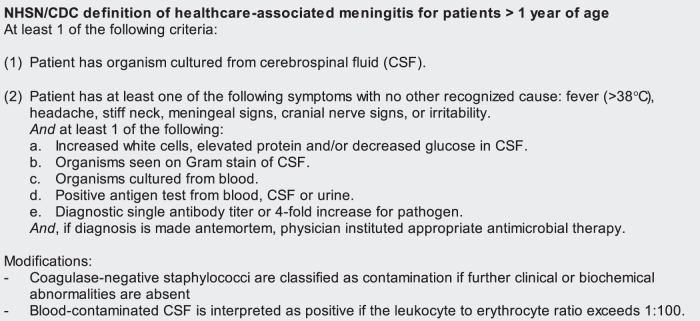
Modified CDC-definition for healthcare-associated meningitis (reference standard).

As in model development, the outcome or reference standard was the development of DRM, which is defined as the occurrence of meningitis when the drain is *in situ* or within seven days of drain removal. Meningitis is defined according to the CDC-definition for healthcare-associated meningitis as applied by the department of hospital hygiene and infection control during routine manual surveillance. Presence of healthcare-associated meningitis requires either a positive culture or a combination of clinical signs, cerebrospinal fluid (CSF) analysis indicative of meningitis and initiation of empiric antimicrobial therapy by the physician. Importantly, this definition for meningitis allows for classification as a meningitis without bacterial growth from microbiological cultures and requires that cultures with skin flora are evaluated for possible contamination (**Figure** **2**) [14]–[16], [26]. All charts were manually reviewed, and possible cases of infection were reviewed by at least two infection control professionals. In case of disagreement, consensus was reached through discussion.

### Model validation and patient population

The previously developed model for the prediction of DRM was validated on an independent cohort of consecutive patients that received an EVD or ELD, selected from the same center though from a later time period (January 2010 to June 11^th^ 2011), a so-called temporal validation [19], [20]. In this time period, surveillance for ELDs was limited to drains placed in operating theatres in 2010 and discontinued in 2011. Data on device utilization is currently collected manually using electronic operating theatre and ICU records. Children (n = 13), patients with a meningitis at drain placement (11), patients who died within 24 hours (5) or who received multiple simultaneous drains (2) and those who were admitted with a drain already in situ (1) were excluded from the analysis, leaving 105 patients in the validation set. Approximately three-quarters of the EVD patients (75 of 99 patients) received a drain due to hydrocephalus after intracranial hemorrhage; nine percent received an EVD to treat increased intracranial pressure caused by a tumor. Five out of six ELD's were placed as a per-operative preventive measure. For each patient in the validation set, the reference standard was determined and predictor data was collected.

### Predictors

Predictors were defined, collected and interpreted as in model development [14]. Predictors were selected for their ability to predict the development of DRM, irrespective of a causal association. Patient characteristics, administrative data (e.g. length of stay, ICU admissions) and the clinical parameters used in the prediction model (**Figure** **1**) were extracted from the clinical data warehouse. Besides the predictors obtained during model development, Gram stain results, the location to which the patient was discharged (i.e. deceased, home or other care facility) and urgency of admission as recorded in administrative files were also available. For each patient, all results obtained throughout the surveillance episode (duration of drainage plus seven days or up to discharge) were retrieved. For each predictor, the value most indicative of infection was used as parameter value; for example, for the peripheral blood leukocyte count the highest value measured during the surveillance episode was entered in the prediction rule.

### Statistical analyses

Missing data were imputed using multiple imputation (10 iterations) to prevent bias that would have occurred if the analysis had been limited to complete cases only [24]. In Table S1, a comparison of cases with and without missing data is given. For model validation, imputation was performed on the validation set only. A new imputation was run on the development and validation set combined for model update (see below). The original model depicted in Figure 1 was validated. Discrimination, classification and prediction at the group level (calibration-in-the-large) were assessed.

### Model updating

Since datasets from both model development and validation were available, we investigated whether the original model could be improved or updated using both datasets combined and hence make maximal use of all data [21], [27]. As opposed to model derivation, patients with multiple simultaneous drains were no longer excluded since they are not expected to be different in terms of diagnosis of DRM. Furthermore, during the updating process, one misclassification error in the model development data was resolved and all data were adapted accordingly thus slightly improving performance characteristics obtained in the model development. All predictors from the original model were included in the revised model to re-estimate their coefficients. In addition, the new predictors (Gram stain, urgency of admission and discharge destination) were added to the model if they significantly improved the model (likelihood ratio test, p-value of 0.05). Gram stain results were combined with CSF culture result and CRP was included as a fractional polynomial to accommodate the non-linear association between CRP and risk of DRM [28]. Estimates were derived from the 10 imputation sets and pooled using Rubin's rule, a method that takes into account variation within and between multiple imputation data sets [25].

Then, internal validation was performed by bootstrapping (100 samples per imputation set, including predictor selection using all predictors considered in model development and update) and a uniform shrinkage factor was applied, this to prevent over-optimism and to make the model generalizable to future patient populations [29]. The final model is presented along with its optimism-corrected performance characteristics. Analyses were performed with SPSS® version 19 (SPSS Inc, Chicago IL) and R version 2.14.1 (www.r-project.org).

## Results

### Model validation

Model validation was performed on 105 patients who received 134 drains. Nearly all patients in the surveillance received an EVD (94.3%), due to discontinuation of ELD surveillance. The infection rate in the validation period was 17.3 per 1000 drainage days at risk (DAR). All infections occurred in patients receiving an EVD. In fifty percent of infections, no positive culture was obtained. Median age in the validation set was 59.3 years (model development 58.5 years), 65.7% of patients were female (model development 54.0%) and 71.5% received a drain to treat hydrocephalus after subarachnoid bleeding, (intraventricular) hemorrhage or infarction (model development 49.0%) and in-hospital mortality after exclusion of patients who died within 24 hours of drain placement was 21.9%. The area under the ROC curve, which is a measure of discrimination, was 0.951 (95% confidence interval (CI) 0.914 to 0.988); during model development an area under the ROC curve of 0.976 (95% CI 0.965–0.987, without correction for optimism) was observed [14]. Calibration-in-the-large, a measure of the total number of infections in a specified time period, predicted 13.46 infections in 2010 (observed  = 13 infections) and 6.06 infections between January 1^st^ and June 10^th^ 2011 (observed  = 7). **Table** **1** gives the contingency table obtained after application of the original prediction model and threshold.

**Table 1:** Contingency table with results of model validation with 95% confidence intervals for sensitivity, specificity and predictive values.

*Predicted probability*	*DRM*				*(%)*	*95% CI*
	Yes	No	Total	Sensitivity	100.0	(83.2–100)
P(DRM) >0.107	20	14	34	Specificity	83.5	(73.9–90.7)
P(DRM) ≤0.107	0	71	71	PPV	58.8	(40.7–75.4)
Total	20	85	105	NPV	100.0	(94.9–100)

Abbreviations: NPV – negative predictive value, PPV – positive predictive value, P(DRM) – predicted probability of drain-related meningitis.

### Model update

The model was updated to incorporate newly available data and optimize performance in new patients. The total 2004–2011 dataset included 653 patients which received 863 drains. The observed infection rate was 14.1/1000 DAR (16.7/1000 DAR for EVDs, 6.0/1000 DAR for ELDs). Baseline characteristics and the results of model re-estimation are presented in **Table** **2**. Patients who developed DRM received multiple courses of antibiotics during their surveillance episode; most likely they suffered from or were suspected of other concomitant infections. The higher mortality in the non-affected group is in part caused by the shorter duration of follow-up in the deceased patients; hence they had less time to develop a DRM.

**Table 2:** Model update results for 2004–2011 data, including baseline characteristics and results of univariable and multivariable analysis.

	Results of univariable analysis			Results of multivariable analysis^b^		
	no DRM	DRM	p-value^a^	Estimate	OR	95% CI
Median (IQR) or n (%)	n = 549	n = 104				
**Baseline characteristics:**						
Age (years)	59.3 (47.3–69.3)	56.1 (47.4–66.4)	0.599			
Sex (% female)	307 (55.9)	58 (55.8)	0.997			
In-hospital mortality (%)	105 (19.5)	12 (11.5)	0.064			
Duration of admission (days)	19 (11–30)	41 (29–63)	<0.001			
ICU admission (%)	322 (58.7)	75 (72.1)	0.010			
**Predictors in previous model:**						
Drain Type (% EVD)	352 (64.1)	93 (89.4)	<0.001	1.49	4.421	1.461–13.373
Number of drains placed	1 (1–1)	2 (1–2)	<0.001	0.52	1.687	1.154–2.698
CRP (mg/L)^c^	99 (37–183)	143 (94–189)	<0.001	−0.08	0.926	0.883–0.972
Peripheral leukocytes (×10^9^/L)	15.3 (11.4–19.4)	20.3 (16.4–24.3)	<0.001	0.08	1.090	1.022–1.153
CSF leukocytes (×100/uL)^d^	1.9 (0.2–6.4)	12.9 (2.7–83.8)	<0.001	0.20	1.224	1.058–1.416
CSF and/or drain culture^e^ (%)	54 (9.8)	77 (74.0)	<0.001			
Any empiric antibiotic therapy (%)	72 (13.1)	81 (77.9)	<0.001	1.80	6.067	2.632–13.983
Number of antibiotics started	1 (0–2)	4 (3–6)	<0.001	0.20	1.225	0.988–1.519
**New variables considered:**						
Emergency admission (%)	312 (56.9)	68 (65.4)	0.109			
Discharge to			<0.001			
– Home	235 (42.8)	25 (24.0)				
– Other (deceased, care facility)	314 (57.2)	79 (76.0)				
CSF and/or drain culture or Gram stain^e^	59 (10.7)	79 (76.0)	<0.001	2.50	12.117	5.202–28.225

a : p-value in univariable analysis by student's t test, Mann-Whitney U test or Chi-square where appropriate.

b : Results of the multivariable analysis are after bootstrapping (shrinkage factor 0.79). The intercept of the model was estimated at –6.615.

c : In the multivariable analysis, all CRP values were divided by factor 10.

d : In the multivariable analysis, CSF leukocytes were log transformed.

e : Culture results corrected for contamination with skin flora; if no antibiotics were started, culture was classified as negative.

Abbreviations: CRP – C-reactive protein; CSF – cerebrospinal fluid; DRM – Drain-related meningitis; EVD – external ventricular drain; OR – Odd's ratio.

In the multivariable analysis, using a fractional polynomial to fit the model to the CRP levels did not lead to the inclusion of higher power terms in the model and only the linear term was retained, albeit with a reversed direction. This is most likely because patients with a very high CRP level suffered from a different infection than DRM. The area under the ROC curve of the updated model was 0.972 before correction for optimism and 0.963 (95% CI 0.953–0.974) after correction for over-optimism. **Table** **3** shows classification results with varying predicted probability cut-offs.

**Table 3:** Model classification results with different predicted probability cut-offs.

P(DRM) cut-off	Sensitivity	Specificity	PPV	NPV	Charts to review
	(%)	(%)	(%)	(%)	(% of total)
0.025	100.0	62.5	33.5	100.0	310 (47.5)
0.050	100.0	78.3	46.6	100.0	223 (34.2)
0.075	99.0	82.7	52.0	99.8	198 (30.3)
0.010	99.0	85.8	56.9	99.8	181 (27.7)
0.125	99.0	86.7	58.5	99.8	176 (27.0)
0.150	98.1	87.6	60.0	99.6	170 (26.0)
0.175	95.2	89.1	62.3	99.0	159 (24.3)
0.200	93.3	90.0	63.8	98.6	152 (23.3)
0.225	90.4	91.1	65.7	98.0	143 (21.9)
0.250	87.5	91.8	66.9	97.5	136 (20.8)
0.275	83.7	93.4	70.7	96.8	123 (18.8)
0.300	82.7	94.2	72.9	96.6	118 (18.1)

With increasing cut-off, the sensitivity decrease is associated with a decrease in number of charts requiring manual review for confirmation of infection.

Abbreviations: DRM – drain-related meningitis, NPV – negative predictive value, PPV – positive predictive value, P(DRM) – predicted probability for drain-related meningitis.

**Figure 3:**
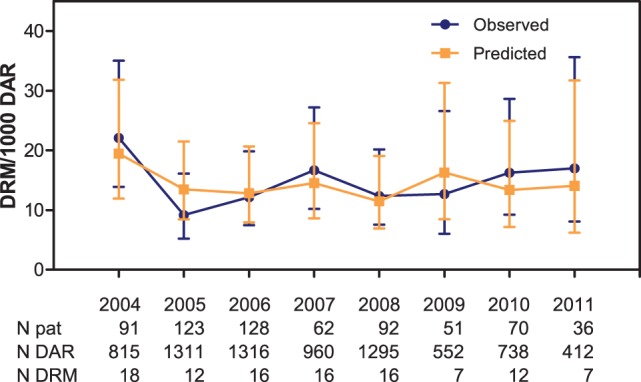
Observed and predicted group-level infection rates using updated model, per 1000 days at risk with 95% confidence intervals. Abbreviations: DRM – drain-related meningitis, DAR – days at risk, N pat – number of patients, N DAR – number of days at risk, N DRM – number of cases of drain-related meningitis.

Finally, yearly infection rates can be estimated by summing predicted probabilities (calibration-in-the-large) for all patients in each year group (**Figure** **3**).

## Discussion

The results of the present study demonstrate that the previously proposed model for the surveillance of DRM, in unaltered form, maintains its high discriminatory power and adequate group-level prediction in a new patient population from the same center. Patients included in the validation set were on average more seriously ill than in the derivation set, probably due to the discontinuation of surveillance of patients receiving ELDs. However, this did not impact model performance. Model update was performed to include predictors that recently became available and optimize the model. Performance of the updated model is similar to the original model. As described in **Table** **3**, a choice needs to be made between sensitivity and specificity in selecting a predicted probability cut-off; increasing the predicted probability cut-off reduces the number of charts to review at the cost of sensitivity. Since this model is applied to patients retrospectively and does not affect clinical decision making, it is worthwhile to, accept a sensitivity of 95.2% as opposed to 100.0% which will reduce the workload for manual review from 223 to 159 charts. As in model development, longitudinal surveillance at the group level can be performed using this model. Comparison of the original and revised model regression coefficients yields similar directions and slightly more conservative magnitudes due to the more stringent model shrinkage procedure used in the model update.

The observed rates of DRM in this study are in the upper part of the spectrum of rates published. The use of a broad definition that includes infections in which no micro-organisms were cultured from CSF may play a role (26% of the infections) [10]. Furthermore, as opposed to benchmarking data from Germany [30], both ICU and non-ICU patients are included in the surveillance and follow-up is extended beyond ICU discharge. The lower infection rate observed in patients who received an ELD (16.7 vs 6.0/1000 DAR) may be explained by the less severe underlying disease in these patients. Schade et al. also found lower DRM rates in patients receiving an ELD as compared to an EVD [31]; as in our population (data not shown), these patients often received an ELD for the prevention or treatment of CSF leakage. In other studies, a higher DRM rate was found in patients receiving ELDs which may be due to the inclusion of almost exclusively patients with underlying intracranial hemorrhage [15].

The updated model presented in this research is, to our knowledge, the only model developed to specifically survey the development of meningitis complicating the use of external CSF drains that has undergone temporal validation. Compared to other automated surveillance systems for (procedure-specific) HAI, this model is one of the few using data from multiple sources in a multivariable model which weights the individual predictors to generate a prediction. This is in contrast to the often seen binary classification algorithms which use fewer data sources and often require positive cultures for case-finding [9], [32], [33]. In this model, positive cultures and antibiotic use are important predictors but no absolute requirement for the detection of infection, thus making it possible to identify those infections in which a positive culture was not obtained or for which the patient was not treated with standard empiric therapy. The study presented here confirms that this multivariable approach is valid for the surveillance of HAI, and may possibly be applied to other infections as well. Currently, use of the model requires extraction of predictor data from the electronic medical records and subsequent data processing prior to application of the prediction rule; ongoing developments in healthcare information technology are expected to facilitate the widespread implementation of such systems.

Since the number of external drains placed on a yearly basis is limited, the validation could only be performed on a relatively small patient population. Therefore, performing multiple imputation on this set of data required very relaxed settings which may cause unstable results. However, the model was subsequently revised and extended using the total population, one of the largest DRM cohorts to date, to make optimal use of available data and return the most reliable model possible. Although model update considered several new variables that have become available in the data warehouse, not all potential risk factors and diagnostic markers of DRM could be included. For example, there is no (field-defined) data on whether the drains were placed during an emergency procedure, how often drains were manipulated or whether there was cerebrospinal fluid leakage at the insertion site [10], [11], [34]. Markers of meningitis under investigation, such as procalcitonin and interleukins [35]–[37], are not routinely determined and thus not included. Furthermore, since the model is dependent on clinical practices, such as culture frequency and antibiotic use policies, the model may need to be adapted when implemented in new settings. However, the model will not be affected by differences in occurrence of causal risk factors assuming that clinical presentation and diagnostic workup remain unaffected. The effect on model performance of differences that may affect clinical presentation, such as use of antibiotic coated catheters, will need to be investigated further. When interpreting the results of this study, it must be realized that it has been developed for the purpose of infection surveillance after the fact, and not for realtime surveillance of infections. Several studies have attempted to identify parameters which can predict the onset of DRM, however with inconclusive results [37], [38]. The current model could be used for more timely feedback of infection rates and may return more consistent results than manual surveillance.

This model for the surveillance of drain-related meningitis has now been temporally validated in a single center, and maintained performance despite small changes in case-mix of the validation set. Multi-center validation is currently ongoing to investigate transportability to other hospitals and validity in patients with a different case-mix; also the effect of the use of antibiotic-coated catheters on model performance will be assessed. Several challenges still remain to achieve implementation in routine surveillance. Methods for handling of missing data in future patients need to be tested, and with the implementation in multiple centers, risk adjustment methods will be necessary to allow for valid comparison between centers. Another aspect that will require attention in the future is quantification of device utilization rates to generate infection rates with reliable numbers both in the numerator (this model) and the denominator.

## Supporting Information

Table S1 Comparison of patients with and without missing data. Complete cases have different underlying disease and are more likely to have developed DRM than non-complete cases.
(DOC)
