# Supplementary material for: Automated Detection of Healthcare Associated Infections: External Validation and Updating of a Model for Surveillance of Drain-Related Meningitis
Source: PLoS One. 2012 Dec 7;7(12):e51509. doi: 10.1371/journal.pone.0051509 (PMC3517564; doi:10.1371/journal.pone.0051509)
Supplement: Table S1 — Comparison of patients with and without missing data. Complete cases have different underlying disease and are more likely to have developed DRM than non-complete cases. (DOC) [file pone.0051509.s001.doc]

**Table S1:** Comparison of patients with and without missing data. Complete cases have different underlying disease and are more likely to have developed DRM than non-complete cases.

| **Predictor** | **complete case^a^** | **non-complete case** |  |
| --- | --- | --- | --- |
| **median or n(%)** | **n = 410** | **n = 243** | **p-value^b^** |
| Age (years) | 58.8 | 57.9 | 0.469 |
| Sex (% female) | 232 (56.6) | 133 (54.7) | 0.645 |
| In-hospital death (%) | 60 (14.6) | 57 (23.5) | 0.004 |
| Duration of admission (days) | 27 | 13 | <0.001 |
| Admission on ICU (%) | 269 (65.6) | 128 (52.7) | 0.001 |
| Indication for first drain (%) |  |  | <0.001 |
| - SAH/IVH/infarction | 259 (63.2) | 101 (41.6) |  |
| - CSF leakage | 51 (12.4) | 36 (14.8) |  |
| - Per-operative | 38 (9.3) | 58 (23.9) |  |
| - Trauma | 12 (2.9) | 4 (1.6) |  |
| - Tumor | 30 (7.3) | 18 (7.4) |  |
| - Other | 38 (9.3) | 26 (10.7) |  |
| Emergency admission (%) | 263 (64.1) | 117 (48.3) | <0.001 |
| DRM (%) | 102 (24.9) | 2 (0.8) | <0.001 |
|  |  |  |  |
| **Drain characteristics** |  |  |  |
| Drain type (% EVD) | 304 (74.1) | 141 (58.0) | <0.001 |
| Total drain duration (days) | 13 | 6 | <0.001 |
| Number of drains placed | 1 | 1 | <0.001 |
|  |  |  |  |
| **Laboratory measures** |  |  |  |
| CRP (mg/L) | 132 | 85 | <0.001 |
| Leukocytes (x 10^9^/L ) | 17.1 | 14.4 | <0.001 |
| CSF Leukocytes (x100/uL) | 1.97 | 0.23 | <0.001 |
| CSF and/or drain culture positive (%) | 119 (29.0) | 8 (7.3) | <0.001 |
| Gram stain positive for bacteria | 71 (17.3) | 3 (4.6) | 0.009 |
|  |  |  |  |
| **Antibiotic use** |  |  |  |
| Any empiric antibiotic therapy (%) | 148 (36.1) | 5 (2.1) | <0.001 |
| Number of antibiotic started | 2 | 0 | <0.001 |

^a^ Data was termed complete if information for all model predictors was available.

^b^: p-values by student’s *t* test, Chi square or Mann-Whitney U test where appropriate

Abbreviations: CRP – C-reactive protein, CSF – cerebrospinal fluid, DRM – drain-related meningitis, EVD – external ventricular drain, ICU – intensive care unit, IVH – intraventricular hemorrhage, SAH – subarachnoid hemorrhage.
